# Anesthesia for stem cell transplantation in autistic children: A prospective, randomized, double-blind comparison of propofol and etomidate following sevoflurane inhalation

**DOI:** 10.3892/etm.2015.2176

**Published:** 2015-01-13

**Authors:** YU-HENG MA, YONG-WANG LI, LI MA, CAI-HONG CAO, XIANG-DONG LIU

**Affiliations:** 1 Department of Anesthesiology, Second Artillery General Hospital of PLA, Beijing 100088, P.R. China; 2 Department of Gynecology and Obstetrics, Second Artillery General Hospital of PLA, Beijing 100088, P.R. China

**Keywords:** sevoflurane, propofol, etomidate, autism, stem cell transplantation

## Abstract

The objective of the present study was to comparatively investigate the feasibility and safety of etomidate and propofol use following sevoflurane inhalation in autistic children during the intrathecal transplantation of stem cells. The patients selected were 60 autistic children with American Society of Anesthesiologists physical status I, who were aged between two and 12 years and scheduled for stem cell transplantation. The children received an inhalation induction of 8% sevoflurane, followed by intravenous injection of etomidate (0.2 mg/kg) in group E and propofol (2 mg/kg) in group P (n=30/group). Supplemental doses of 0.1 mg/kg etomidate or 1 mg/kg propofol were used until a deep sedation was obtained. The heart rate (HR), mean arterial pressure, oxygen saturation, respiratory rate, Ramsay sedation score (RSS) and recovery time were monitored continuously. Following anesthesia, blood pressure and HR measurements were significantly decreased in group P compared with the baseline (P<0.01) and group E values at the same time-points (P<0.05). The occurrence of adverse effects, such as respiratory depression, bradycardia, hypotension and pain on injection, was significantly higher in group P than that in group E, whereas the incidence of myoclonus in group E was significantly higher than that in group P (P<0.01). No significant differences in anesthesia induction, surgery duration, recovery time, RSS and physician satisfaction were observed between the two groups. In conclusion, sevoflurane-etomidate combinations resulted in more stable hemodynamic responses and relatively fewer adverse effects compared with propofol injection following sevoflurane inhalation and may therefore be more suitable for the induction of short-term anesthesia in autistic children during stem cell transplantation.

## Introduction

Autism and autism spectrum disorder (ASD) are frequent and severe developmental disorders of the central nervous system, characterized by dysfunctional social interactions and communication skills, along with repetitive and stereotypical verbal and nonverbal behaviors (1). The etiology of ASD remains unclear; however, the condition most likely results from a complex combination of genetic, environmental and immunological factors (2,3). Although prescription drugs and education may reduce some symptoms of autism (4), there is currently no cure available for ASD. Stem cell transplantation via subarachnoid cavity injection is reported to be a novel, promising treatment for specific types of autistic children (5). Although lumbar puncture is a safe and relatively short procedure, the patients are required to remain motionless during the process. Autistic children with cognitive handicaps are unable to cooperate with this request and present unique challenges to the medical team; therefore, a rapid and effective induction of anesthesia is indispensable for autistic patients during the lumbar puncture procedure.

Sevoflurane, a novel type of inhalation anesthetic with rapid induction and fine control ability, is non-pungent and non-irritating to the respiratory tract (6). Sevoflurane can be inhaled using a face mask, thus making it suitable for short procedures and for use in patients with venipuncture difficulties. Alternatively, as short-acting, intravenous anesthetics, propofol and etomidate have been found to have a rapid onset time and short duration (7,8). These agents are typically used for the induction of general anesthesia and for sedation during short procedures, such as gastroenterological endoscopy, cervix examination or tracheal intubation (9–12). Several studies have revealed that, when compared with propofol, etomidate did not decrease or only slightly decreased arterial blood pressure and cardiac output during the induction of anesthesia (9,13), indicating a lower risk of cardiovascular depression.

In the present study, we hypothesized that etomidate administration would achieve a greater hemodynamic stability and less respiratory depression when used as an anesthetic in autistic children. The purpose of this clinical, prospective, randomized, double-blind study was to comparatively study the efficacy and adverse effects of two anesthetics, propofol and etomidate, administered by injection, in order to determine which, if any, would be suitable for the induction of short-term anesthesia in autistic children during stem cell transplantation, when used in combination with sevoflurane inhalation.

## Materials and methods

### General approach and patient cohort

The study protocol was reviewed and approved by the Ethics Committee of the Second Artillery General Hospital of PLA (Beijing, China), and written informed consent was obtained from the subjects, and parents of minors. A total of 60 autistic children with American Society of Anesthesiologists physical status I, aged 2–12 years and scheduled for stem cell transplantation via lumbar puncture during January 2011 to October 2012, were recruited in the study. The enrolled patients (51 males and nine females) were randomly allocated equally to the propofol group (group P) and the etomidate group (group E). Exclusion criteria consisted of a history of epilepsy, necessary use of central nervous system medications prior to or following the surgery, an allergy to the anesthetics used in this study and evidence of corticoadrenal insufficiency.

Propofol and etomidate are both opaque white liquids, which allowed the study to be conducted under double-blind conditions. The recommended dose of etomidate is between 0.15 and 0.3 mg/kg and that of propofol is between 1.5 and 2.5 mg/kg (7,8). In the present study, the patients received either 0.2 mg/kg intravenous etomidate or 2 mg/kg intravenous propofol according to their group assignment.

### Anesthesia procedure

Patients were fasted for 6 h and water-deprived for 4 h prior to the surgery, and no premedication was administered. The general anesthesia procedure was as follows: First, the patients received an inhalation induction with 8% sevoflurane (Jiangsu Hengrui Medicine Co., Ltd., Shanghai, China) at an oxygen flow of 3 l/min. Once the patients had fallen asleep, the face mask was rapidly removed and standard anesthesia monitoring and peripheral venous access were quickly established. Each child was intravenously injected with 2.0 mg dexamethasone and 0.01 mg/kg scopolamine; then 0.2 mg/kg etomidate (Jiangsu Nhwa Pharmaceutical Ltd. by Share Ltd., Xuzhou, China) was intravenously administered to group E patients and 2 mg/kg propofol (AstraZeneca, London, UK) to group P. Supplemental doses of 0.1 mg/kg etomidate in group E and 1 mg/kg propofol in group P were repeatedly given if sedation was not adequate, as defined by a Ramsay sedation score (RSS) (14) of <4. Anesthetic drugs were administered slowly, ≥30 sec.

### Stem cell transplantation

Stem cell transplantation was performed with the patients oriented in a lateral decubitus position to maximize the spinal interspaces for lumbar punctures. The puncture areas were infiltrated with 1 ml 0.5% lidocaine. Routine lumbar punctures were performed by the same physician and cerebrospinal fluids were collected in aseptic test tubes for testing. Stem cells were then injected into the subarachnoid space. During the procedure, the patients were given oxygen at a flow rate of 3 l/min using a face mask.

### Observation indices

Data from clinical parameters, such as heart rate (HR), mean arterial pressure (MAP), pulse oxygen saturation (SpO_2_), respiratory rate (RR), RSS and recovery time (time to reach an RSS of 2), were continuously monitored throughout the procedure. Adverse effects, such as respiratory depression (RR <8 breaths/min), hypotension (decrease in MAP >30% from the baseline), hypertension (increase in MAP >30% from the baseline), arrhythmia, bradycardia (HR <60 bpm) and hypoxemia (SpO_2_ <95%) were recorded. Oxygen was supplied for each patient throughout the surgery, and their physician satisfaction was evaluated using an objective four-point scale (very good, good, fair or poor). Discrete time-points included study onset (T0), the administration of the propofol or etomidate (T1), and 5 min after the administration of the propofol or etomidate (T_5min_).

### Statistical analysis

Statistical analyses were performed using SPSS software (version 13; SPSS Inc., Chicago, IL, USA). Continuous and discrete variables are expressed as the mean ± standard deviation and the numbers of patients, respectively. Data were analyzed using the independent samples t-test, repeated-measures analysis of variance or least significant difference t-test when appropriate. Numeration data were analyzed using a χ^2^ test. P<0.05 and P<0.01 were considered to indicate statistically significant and highly statistically significant differences, respectively.

## Results

### Physical characteristics and cardiovascular indices

**Figure 1:**
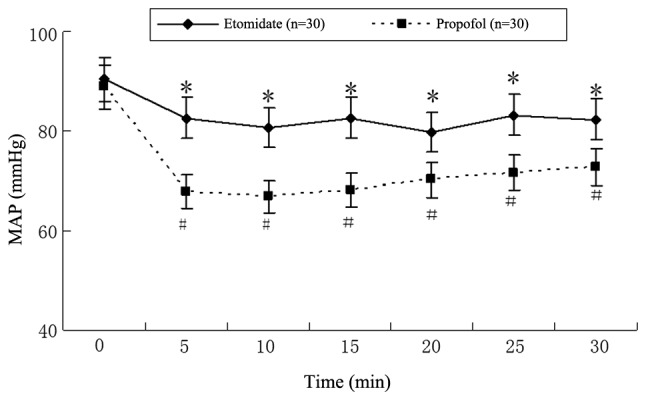
Effect of propofol and etomidate injection following sevoflurane inhalation on MAP. Following anesthesia, blood pressure measurements were decreased in groups E and P compared with the baseline values, and the MAP level in group P was significantly lower than that in group E at each time-point (P<0.05). ^#^P<0.01 vs. baseline; ^*^P<0.05 vs. group P at the same time-point. MAP, mean arterial pressure; group E, etomidate group; group P, propofol group.

**Figure 2:**
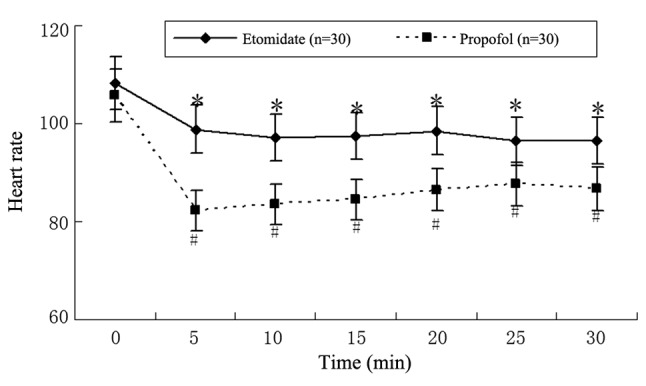
Effect of propofol and etomidate injection following sevoflurane inhalation on HR. Following anesthesia, HR decreased in both groups E and P compared with the baseline values. The HRs in group P were significantly lower than those in group E at the same time-point (P<0.05). ^#^P<0.01 vs. the baseline; ^*^P<0.05 vs. group P at the same time-point. HR, heart rate; group E, etomidate group; group P, propofol group.

The physical characteristics of the patients were comparable in the two groups, and no statistically significant differences were found in age, gender, weight or physical status (P>0.05, Table I). Following anesthesia, blood pressure and HR were decreased in groups E and P compared with the baseline values (Figs. 1 and 2), but the levels were significantly lower in group P than those in group E (P<0.05). The MAP and HR of patients in group P significantly decreased during the first 5-min interval (88.9±5.7 mmHg and 105.7±3.7 bpm at T1 to 67.9±3.9 mmHg and 82.3±8.7 bpm at T_5min_, respectively; P<0.01). The same tendency existed in group E patients, where MAP and HR were respectively decreased from 90.4±4.2 mmHg and 108.3±4.8 bpm at T1 to 82.6±3.6 mmHg and 98.8±7.8 bpm at T_5min_ following anesthesia; however, this difference did not reach statistical significance (P>0.05).

During the procedure a decrease in SpO_2_ was observed within each group. The SpO_2_ in group E was reduced from 98.7±0.7% at T1 to 96.8±0.8% at T_5min_ (P<0.05), while propofol produced a larger decrease in SpO_2_ from 98.6±0.8% at T1 to 95.8±0.9% at T_5min_ (P<0.05). Due to the oxygen supply during the entire procedure, however, no significant difference in SpO_2_ was observed between the two groups.

### Adverse effects

The adverse effects caused by anesthesia are summarized in Table II. The results showed that the episodes of respiratory depression, bradycardia, hypotension and pain on injection of propofol were significantly higher than the number of adverse episodes identified in the etomidate-treated group (P<0.05). By contrast, myoclonus was estimated to occur in 26.7% of the children anesthetized by etomidate, while no equivalent symptom was identified in group P patients (P<0.01). For the analysis of physician satisfaction, no significant difference was identified between the two groups (Table I).

### Surgery parameters

No statistically significant differences were found between groups E and P with respect to the length of anesthesia induction (the time from the start of sevoflurane inhalation to the onset of sleep), surgery duration and recovery time (P>0.05, Table I). Similarly, no significant difference was observed with regard to the RSSs between the two groups, indicating that propofol and etomidate may achieve similar levels of sedation within 20 min of the injection of the anesthetics (Table III).

## Discussion

Autism is a complex developmental disability that is typically observed during childhood and that may result from a neurological disorder that affects the functioning of the human brain. Children diagnosed with ASD are often characterized by significant deficits in reciprocal social interaction, verbal and nonverbal communication skills and imaginative activity. As a result of their highly unusual clinical presentation, autistic children present a unique perioperative challenge to the pediatric surgical team (15); therefore, stem cell transplantation for children with autism must be performed under general anesthesia.

The main goals of anesthesia are to establish a rapid recovery, with reduced postoperative pain, few complications, and minimal respiration and circulatory system changes during the perioperative period. As short-acting intravenous anesthetics, propofol and etomidate have been used for the induction and maintenance of anesthesia in a rapid and safe manner (16–18). Thus, in the present study, the efficacy and adverse effects of these two anesthetics were comparatively studied in 60 autistic children during stem cell transplantation, to find which anesthetic would be suitable for induction of short-term anesthesia in autistic children.

The results presented showed that, unlike propofol, which markedly suppressed the circulatory system, etomidate administration only slightly reduced the blood pressure and HR of the pediatric patients. This was unsurprising since etomidate has been reported to neither selectively decrease sympathetic nervous system activity nor inhibit cardiovascular function (7,8). Previous studies have also suggested that etomidate has minimal effects on healthy patients or those with cardiac diseases, making it more suitable for patients with hypotension, hypovolemia or cardiovascular disorders (8,19). By contrast, propofol has been reported to clearly inhibit sympathetic nerve activity and baroreceptor reflexes (20), thereby making it more likely to be associated with bradycardia and hypotension (19). In the current study, bradycardia occurred in five children in group P during their recovery, while no bradycardia occurred in group E children, which corroborated the results of previous studies.

Another serious problem associated with the use of propofol is reputed to be the high incidence of pain at the injection site (25–50%) (8) In the present study, a total of five children receiving a propofol infusion complained about pain on injection, while no one developed this complication following etomidate administration. In addition, etomidate has previously resulted in less apnea or respiratory depression when compared with propofol (21). Due to the continuous oxygen supply, however, no significant difference in SpO_2_ was detected between the two groups; therefore, the same conclusion could not be drawn from the present study.

The most prominent adverse effect reported with etomidate was myoclonus, which has been suggested to be dose-dependent and to occur in 20–45% of patients (22). The findings of the current study showed a 26.7% incidence of myoclonus in group E children while under anesthesia; the practice of slow etomidate administration may explain this lower incidence found.

A major concern associated with etomidate infusion is the possibility of adrenal suppression (23,24). Etomidate has been previously found to inhibit 11-β-hydroxylase, an enzyme that promotes the conversion of 11-deoxycortisol to cortisol (25), and long-term, high-dosage use of etomidate may cause adrenal suppression. While it was conceivable that the short-term use of etomidate was not likely to lead to adrenocortical suppression (8), in the present study, although adrenocortical function was not directly measured, certain outward signs of adrenal suppression, including hypotension and arrhythmia, were monitored. The results showed that there was not any outward sign of adrenal suppression identified following etomidate infusion.

In addition to myoclonus and adrenal suppression, postoperative nausea and vomiting are frequent side effects of etomidate. A previous study reported a post-operative nausea and vomiting incidence of 25–30% following etomidate induction, higher than that when propofol was used (9); however, in the current study, no children developed nausea or vomiting following etomidate administration. This was perhaps due to dexamethasone infusion prior to the procedure, which may have inhibited the development of nausea and vomiting (26).

No statistically significant difference was found between group E and P patients with respect to the length of anesthesia induction, surgery duration and recovery time (P>0.05). The RSS, a tool for measuring the quality of sedation in patients, was also evaluated in this study, with no significant difference observed between the two groups. This indicated that propofol and etomidate may achieve similar levels of sedation within 20 min after intravenous injection.

In conclusion, the results of the present study suggested that a sevoflurane-etomidate combination achieved a more stable hemodynamic response and resulted in fewer adverse effects compared with a sevoflurane-propofol combination. The sevoflurane-etomidate combination would therefore be more suitable for the induction of short-term anesthesia in autistic children during stem cell transplantation.

## Figures and Tables

**Table I:** General comparison between group E and group P patients.

	Group E, n=30	Group P, n=30
Demographic data		
Age (years)^a^	4.66±2.28 (2–12)	4.49±2.88 (2–12)
Body weight (kg)^a^	19.58±8.97 (15–48)	20.11±9.08 (16–45)
Gender (male/female)	25/5	26/4
ASA	I	I
Physician satisfaction scores		
Very good	20	22
Good	9	8
Fair	1	0
Poor	0	0
Clinical data		
Etomidate total dose (mg/kg)^b^	0.34±0.11	0
Propofol total dose (mg/kg)^b^	0	2.20±0.48
Anesthesia duration (min)^b^,^c^	1.50±0.35	1.60±0.28
Surgery duration (min)^b^	9.66±2.56	9.42±2.54
Recovery time (min)^b^	6.80±2.38	7.20±2.05

a Presented as the mean ± SD (range);

b presented as the mean ± SD.

c The time from the start of sevoflurane inhalation to the onset of sleep.

SD, standard deviation; group E, etomidate group; group P, propofol group; ASA, American Society of Anesthesiologists.

**Table II:** Adverse effects of propofol or etomidate injection following sevoflurane inhalation.

	Group E, n=30	Group P, n=30	P-value
Respiratory depression	5	15	0.006
Nausea and vomiting	0	0	
Bradycardia	0	5	0.019
Hypotension	0	12	0.0001
Myoclonus	8	0	0.002
Pain at injection site	0	5	0.019

Group E, etomidate group; group P, propofol group.

**Table III:** Comparison of Ramsay sedation scores between patients in groups E and P.

Time (min)	Group E, n=30	Group P, n=30
0	1.00±0.00	1.00±0.00
2	5.03±0.88	5.11±0.38
4	5.53±0.68	5.67±0.44
6	5.36±0.67	5.48±0.42
8	5.33±0.75	5.42±0.33
10	5.07±0.86	5.12±0.77
12	3.50±1.14	3.60±1.25
14	2.53±0.73	2.55±1.01
16	1.83±0.59	1.82±0.89
18	1.43±0.63	1.49±0.65
20	1.27±0.45	1.25±0.66

Values are presented as the mean ± standard deviation. Group E, etomidate group; group P, propofol group.
